# EEG Biomarkers for Alzheimer's Disease: A Novel Automated Pipeline for Detecting and Monitoring Disease Progression

**DOI:** 10.1002/alz70856_096963

**Published:** 2025-12-24

**Authors:** Leif ER Simmatis, Emma E Russo, Tayo Steininger, Haleigh Riddell, Evelyn Chen, Queenny Chiu, Michelle Lin, Donghun Oh, Porsha Taheri, Irene E Harmsen, Nardin Samuel

**Affiliations:** ^1^ Cove Neurosciences, Toronto, ON, Canada; ^2^ University of Toronto, Toronto, ON, Canada

## Abstract

**Background:**

Alzheimer's disease (AD) is a neurodegenerative disorder that profoundly alters brain function and organization. Currently, there is a lack of validated functional biomarkers to aid in diagnosing and classifying AD. Therefore, there is a pressing need for early, accurate, non‐invasive, and accessible methods to detect and characterize disease progression. Electroencephalography (EEG) has emerged as a minimally invasive technique to quantify functional changes in neural activity associated with AD. However, challenges such as poor signal‐to‐noise ratio – particularly for resting‐state (rsEEG) recordings – and issues with standardization have hindered its broader application.

**Method:**

To address these challenges, we conducted a pilot analysis of our custom automated preprocessing and feature extraction pipeline to identify indicators of AD and correlates of disease progression.

**Results:**

Our pipeline successfully detected several new and previously established EEG‐based measures indicative of AD status and progression, demonstrating strong external validity.

**Conclusion:**

Our findings suggest that this automated approach provides a promising initial framework for implementing EEG biomarkers in the AD patient population, paving the way for improved diagnostic and monitoring strategies.

